# Deregulated miRNAs in Hereditary Breast Cancer Revealed a Role for miR-30c in Regulating KRAS Oncogene

**DOI:** 10.1371/journal.pone.0038847

**Published:** 2012-06-11

**Authors:** Miljana Tanic, Kira Yanowsky, Cristina Rodriguez-Antona, Raquel Andrés, Iván Márquez-Rodas, Ana Osorio, Javier Benitez, Beatriz Martinez-Delgado

**Affiliations:** 1 Human Genetics Group, Spanish National Cancer Research Centre (CNIO), Madrid, Spain; 2 Centro de Investigación Biomédica en Red de Enfermedades Raras (CIBERER), Madrid, Spain; 3 Hereditary Endocrine Cancer Group, Spanish National Cancer Research Centre (CNIO), Madrid, Spain; 4 Medical Oncology Service, Hospital Clinico Universitario Lozano Blesa, Zaragoza, Spain; 5 Medical Oncology Service, Hospital Gregorio Marañón, Madrid, Spain; University of Nebraska Medical Center, United States of America

## Abstract

Aberrant miRNA expression has been previously established in breast cancer and has clinical relevance. However, no studies so far have defined miRNAs deregulated in hereditary breast tumors. In this study we investigated the role of miRNAs in hereditary breast tumors comparing with normal breast tissue. Global miRNA expression profiling using Exiqon microarrays was performed on 22 hereditary breast tumors and 15 non-tumoral breast tissues. We identified 19 miRNAs differentially expressed, most of them down-regulated in tumors. An important proportion of deregulated miRNAs in hereditary tumors were previously identified commonly deregulated in sporadic breast tumors. Under-expression of these miRNAs was validated by qRT-PCR in additional 18 sporadic breast tumors and their normal breast tissue counterparts. Pathway enrichment analysis revealed that deregulated miRNAs collectively targeted a number of genes belonging to signaling pathways such as MAPK, ErbB, mTOR, and those regulating cell motility or adhesion. *In silico* prediction detected KRAS oncogene as target of several deregulated miRNAs. In particular, we experimentally validated KRAS as a miR-30c target. Luciferase assays confirmed that miR-30c binds the 3′UTR of KRAS transcripts and expression of pre-miR-30c down-regulated KRAS mRNA and protein. Furthermore, miR-30c overexpression inhibited proliferation of breast cancer cells. Our results identify miRNAs associated to hereditary breast cancer, as well as miRNAs commonly miss-expressed in hereditary and sporadic tumors, suggesting common underlying mechanisms of tumor progression. In addition, we provide evidence that KRAS is a target of miR-30c, and that this miRNA suppresses breast cancer cell growth potentially through inhibition of KRAS signaling.

## Introduction

Breast cancer is the most common malignancy among women in developed countries. The majority of breast cancers are sporadic, while familial breast cancer comprises 5–10% of all breast cancers. Germline mutations in the currently known high risk-breast cancer genes (such as BRCA1/2) are common in familial breast cancer, but they can explain, at best, 20–25% of the overall excess familial risk. [1]. Still, the large majority of breast cancer cases that arise in families with strong familial aggregation are not explained by mutations in any know breast cancer susceptibility gene, and are designated as BRCAX-type tumors [2].

In the past decade, gene expression profiling by microarray analysis has lead to great advances in classification of human breast tumors, and the identification of five reproducible molecular subtypes of breast cancer, that have distinct biological features, clinical outcomes, and responses to chemotherapy [3]. On the other hand, there have been only a handful of studies focused on familial breast cancer, due to difficulties in collecting the tumor material, demonstrating that BRCA1/2-mutated breast tumors could be distinguished from sporadic ones based on their gene expression signatures [4].

Recently, microRNA (miRNA) expression profiling calls a great attention to define various types of cancers [5], [6]. miRNAs are an abundant class of small ∼22 nt long single-stranded non-coding RNA molecules acting as negative regulators at post-transcriptional level by binding the 3′ untranslated regions (3′UTRs) of their mRNA-targets [7]. miRNAs are involved in crucial biological processes including development, differentiation, apoptosis and proliferation [6]. Notably, miRNA deregulation has been extensively implicated in cancer pathogenesis in various tumor types [8], [9]. The observed effects of miRNA mis-expression on tumor initiation, maintenance or metastasis can be explained by the mRNA targets and pathways they regulate, which include known tumor suppressors and oncogenes [10]. Specifically, in breast cancer, various studies have identified mis-expressed miRNAs in tumours vs. normal tissue, and shown that changes in their expression seem to define, similarly to what has been found by expression profiling of coding genes, different histological (lobular/ductal, ER+/ER−) [11], [12] and molecular (luminal A, luminal B, basal-like, HER2+) subtypes described so far [13]. In addition, integration of miRNA and mRNA data of a set of breast cancer samples allowed the association of miRNAs to relevant cellular processes, such as proliferation, cell cycle, immune response or cell adhesion, as well as with molecular characteristics of tumors like TP53 mutations [14].

Still, very little is known about the role of miRNAs in familial breast cancer. The identification of target genes and pathways regulated by miRNAs would be critical to understand their function in tumor development. In this study we sought to establish miRNA expression profiles using microarray technology of familial breast cancer tumors and comparing with normal breast tissues. Interestingly, KRAS has been identified as a target oncogene for down-regulated miRNAs. Direct regulation of KRAS by miR-30c and growth inhibition by this miRNA was experimentally demonstrated. The identification of miRNAs deregulated in familial breast tumors could provide a better understanding of the biology of familial breast cancer and could indicate novel targets for therapy.

## Results

### miRNA Expression Profiling in Primary Familial Breast Tumors and Normal Breast Tissue

**Figure 1:**
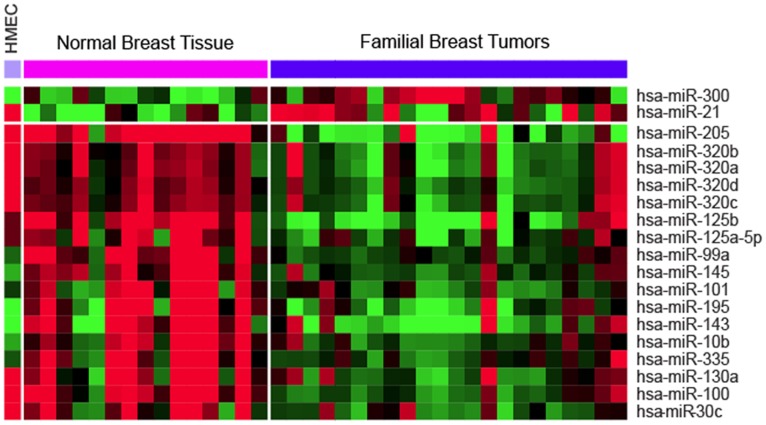
Differentially expressed miRNAs between normal breast tissue and hereditary breast tumors. Heat map of the expression of the 19 miRNAs differentially expressed between normal samples and tumors, overexpression in red, lower expression in green. Expression of these miRNAs in Human Mammary Epithelial Cells (HMEC) cells is also represented.

In order to establish the miRNA profile of hereditary breast tumors, we used LNA based microRNA microarrays. After initial preprocessing we had data from 1276 hsa-miRNAs (831 hsa-miR and 434 hsa-miRPlus). A filter procedure to eliminate genes with low expression variation across the experiments (SD<0.3) and with uniformly low expression, reduced the number of miRNAs to a total of 327 hsa-miRNAs (198 hsa-miR and 118 miRPlus). In an effort to detect significant differences in miRNA expression between normal breast tissue and hereditary breast tumors, we performed a differential expression analysis. We identified 19 miRNA significantly differentially expressed (FDR<0.05) between normal breast tissue and familial tumoral samples (Figure 1). Almost all differentially expressed miRNAs were found to be down-regulated in tumor tissues, with the exception of miR-21 and miR-300 that were up-regulated in breast tumors compared to normal breast tissue (Table 1). Down-regulation of selected miRNAs was validated by qRT-PCR in an independent set of tumors (Figure S1).

**Table 1:** miRNAs differentially expressed between normal breast and familial tumor tissue.

miRNA	Chromosomal location	Median NormalBreast	Median TumorTissue	Fold change	Unadjusted p value	FDR^1^
hsa-miR-205	1q32.2	9.9	7.4	5.9 ↓	3.00E−07	7.82E−05
hsa-miR-125b	11q24.1/21q11.2	11.4	8.9	5.8 ↓	6.00E−07	7.82E−05
hsa-miR-99a	21q11.2	7.4	6.3	2.2 ↓	1.30E−06	0.0001
hsa-miR-100	11q24.1	7.6	6.4	2.2 ↓	2.00E−06	0.0001
hsa-miR-145	5q32−33	7.3	6.4	1.8 ↓	4.95E−05	0.0024
hsa-miR-195	17p13	9.1	7.4	3.4 ↓	0.000152	0.0062
hsa-miR-10b	2q31	7.6	6.8	1.7 ↓	0.00024	0.0084
hsa-miR-320c	18q11.2	7.6	6.9	1.6 ↓	0.000412	0.0127
hsa-miR-320d	13q14.11/Xq27.1	7.4	6.7	1.6 ↓	0.000512	0.0140
hsa-miR-101	1p31.3	7.4	6.4	2.0 ↓	0.000787	0.0185
hsa-miR-130a	11q12	7.5	6.8	1.6 ↓	0.000887	0.0185
hsa-miR-320b	1p13.1/1q42.11	8.1	7.3	1.8 ↓	0.000938	0.0185
hsa-miR-125a-5p	19q13.4	8.8	8.1	1.6 ↓	0.000976	0.0185
hsa-miR-335	7q32.2	7.1	6.5	1.4 ↓	0.001195	0.0210
hsa-miR-320a	8p21.3	8.1	7.3	1.8 ↓	0.001669	0.0257
hsa-miR-143	5q32−33	9.7	8.2	2.8 ↓	0.002078	0.0301
hsa-miR-21	17q23.1	9.1	10.1	2.0 ↑	0.003822	0.0495
hsa-miR-30c	6q13	8.4	7.8	1.5 ↓	0.004283	0.0504
hsa-miR-300	14q32.31	7.1	8.0	2.0 ↑	0.004306	0.0504

1 FDR: False discovery rate adjusted p value.

Expression of these 19 differentially expressed miRNAs in normal Human Mammary Epithelial Cells (HMEC) was similar to the expression in normal breast tissue although some differences also exist for specific miRNAs. HMEC cells represent normal proliferating cells and normal tissue represent preferentially non proliferating cells, therefore, down regulation of some miRNAs, such as miR-99a, miR-101 or miR-145 (Figure 1), might be related to both normal and tumoral proliferation. However, down-regulation of other miRNAs, miR-205, miR-125a/b, miR-100 or miR-30c, might have a role in more specific tumoral processes.

### miRNAs Commonly Deregulated in Familial and Sporadic Breast Cancer

**Figure 2:**
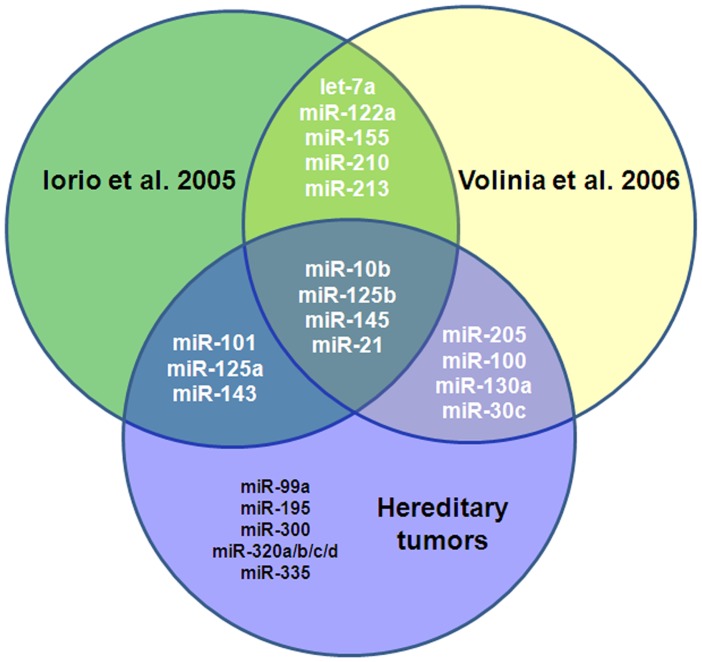
Shared differentially expressed miRNAs in sporadic breast tumors and hereditary tumors. Venn diagrams representing commonly deregulated miRNAs in two different studies carried out in sporadic breast cancer samples [11], [12] and in the present study on hereditary breast tumors. Regardless of the genetic background or histopathological features of the tumors, there are miRNAs consistently altered in breast tumor samples.

Interestingly, several miRNAs that we found to be deregulated in familial breast cancer were previously described to be deregulated in sporadic breast tumors. Thus, 11 of the 19 miRNAs (miR-10b, -100, -101, -125a, -125b, 130a, -143, -145, -21, -205, and -30c) were previously identified in two key studies as being miss-regulated in sporadic breast tumors in comparison to normal tissue [11], [12], suggesting that these miRNAs may play a general role in breast carcinogenesis (Figure 2). Deregulation of some of these miRNAs in sporadic tumors was also confirmed by qRT-PCR analysis in an independent set of 18 sporadic breast tumors (Figure S1).

### Pathway Enrichment Analysis

Given the fact that a single miRNA can target a large number of mRNA transcripts, miss-expression of a set of miRNAs could have significant effect on cellular function by affecting multiple signaling pathways. To assess the potential impact of deregulated miRNA in hereditary breast tumors on biological processes and pathways, we used Diana miRPath web-based computational tool for biological interpretation of miRNA profiling data using over-representation analysis of biological processes and signaling pathways that are targeted collectively by co-expressed miRNAs. KEGG pathway enrichment analysis has revealed that the set of differentially expressed miRNAs between normal tissue and hereditary breast tumors, targets multiple effectors of pathways involved in ubiquitilation, cell proliferation and migration. Full list of pathways that have significantly overrepresented genes (p<0.05) collectively targeted by the set of 19 differentially expressed miRNAs is shown in Table 2. Observed down regulation of miRNAs in tumors regulating expression of these genes may result in abnormally activated pathways leading to increased proliferation and/or migration abilities.

**Table 2:** Significantly enriched signaling pathways associated to the differentially expressed. miRNAs.

KEGG Pathway	N° of miRNA Target Genes	-LN(p-value)	Gene Names
**Ubiquitin mediated proteolysis**	20	17.18	UBE2D1, SOCS1, UBE2D2, UBE3C, UBE1, MAP3K1, BIRC6, UBE2J1, UBE2I, SMURF1, UBE2W, CBL, BTRC, WWP1, CUL2, SOCS3, CBLB, NEDD4L, NEDD4, ITCH
**Axon guidance**	17	11.88	SRGAP3, PLXNA2, GNAI2, DPYSL2, ITGB1, UNC5C, EFNA3, SEMA4D, EPHB2, SEMA6D, KRAS, CFL2, NRP1, PPP3CA, RASA1, SEMA3A, NFAT5
**Insulin signaling pathway**	16	7.96	PPARGC1A, SOCS1, MAPK8, TSC1, PDPK1, KRAS, CBL, FRAP1, CRKL, SORBS1, SOS1, FOXO1, SOCS3, CBLB, PIK3CD, AKT3
**O-Glycan biosynthesis**	6	7.23	GALNT2, GALNT7, GALNT1, GALNT3, GCNT1, B4GALT5
**mTOR signaling pathway**	7	5.31	TSC1, PDPK1, FRAP1, ENSG00000164327, RPS6KA3, PIK3CD, AKT3
**ErbB signaling pathway**	10	4.74	MAPK8, NRG3, KRAS, CBL, FRAP1, CRKL, SOS1, CBLB, PIK3CD, AKT3
**Glycan structures - biosynthesis 1**	12	4.35	GALNT2, GALNT7, GALNT1, GALNT3, CHST2, STT3B, CHST1, GCNT1, EXTL2, B4GALT5, MAN1A2, XYLT1
**MAPK signaling pathway**	20	3.84	MAP4K4, MAPK8, MAP3K1, KRAS, MEF2C, BDNF, CRKL, TAOK1, STK4, SOS1, FGFR1, RPS6KA5, FGF2, RAP1B, PPP3CA, NF1, RASA1, MAP3K12, RPS6KA3, AKT3
**Regulation of actin cytoskeleton**	17	3.61	ITGB1, WASL, ARHGEF6, KRAS, PIP4K2B, CRKL, CFL2, PIP4K2A, ITGA6, SOS1, FGFR1, FGF2, ITGB3, GNA13, ACTC1, PIK3CD, PFN2
**Adherents junction**	8	3.55	IGF1R, SNAI1, WASL, SMAD2, SORBS1, FGFR1, SSX2IP, PVRL1
**Focal adhesion**	16	3.54	BCL2, MAPK8, ITGB1, IGF1R, PDPK1, CRKL, ITGA6, SOS1, PTEN,PTENP1, RAP1B, ITGB3, ARHGAP5, CCND2, PIK3CD, AKT3, CCND1
**T cell receptor signaling pathway**	9	3.2	KRAS, CBL, SOS1, PPP3CA, CBLB, PDCD1, NFAT5, PIK3CD, AKT3

**Figure 3:**
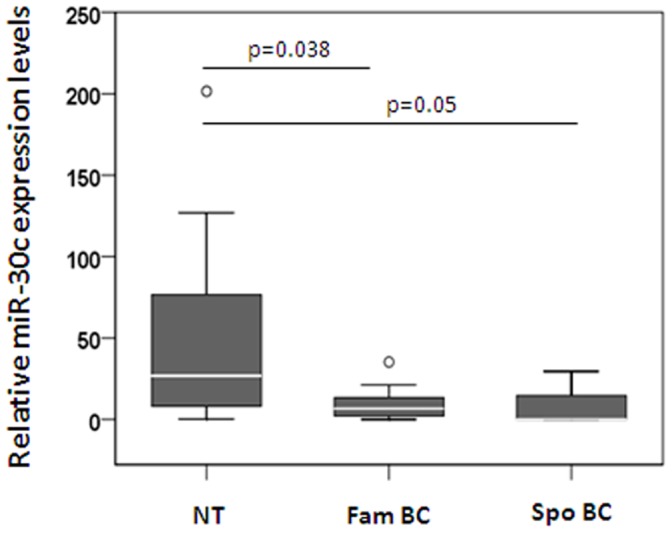
Validation of miR-30c expression by qRT-PCR in hereditary and sporadic tumors. Expression levels of miR-30c in an independent set of 12 familial (FamBC) and 8 sporadic tumors (SpoBC) comparing to normal breast tissue expression. Differences were estimated by t-test and *p* values are shown for each comparison.

We focused on the MAPK signaling pathway since a large number of genes within this pathway were found to be commonly targeted by 8 out of 19 deregulated miRNAs in our study suggesting that these miRNAs might cooperate to affect gene expression and consequentially activation or repression of signaling pathways (Table 2). Interestingly, miR-30c has potential binding sites on 20 different target genes involved in MAPK pathway (Table S1). This miRNA potentially target important mediators of MAPK signaling, such as KRAS, RASA1, MAP3K1 and MAPK8. Furthermore, KRAS gene has been previously validated as a target of several miRNAs, including let-7, miR-143 and miR-96 [15], [16], [17]. Now, we investigated whether miR-30c could be regulating KRAS expression in hereditary breast tumors. Thus, we confirmed by pRT-PCR that miR-30c had decreased expression in both hereditary and sporadic breast tumors comparing to normal samples (Figure 3). In addition, the other KRAS regulating miRNAs, let-7 and miRNA-143 were previously found to be significantly down-regulated in breast tumors [15], [16], while miR-96 regulated KRAS in pancreatic tumors [17]. Although mutations in KRAS are infrequent in breast tumors, activation of KRAS pathway in breast cancer have been frequently found. All these data suggests that deregulation of miRNAs would be a mechanism to explain KRAS overexpression in breast tumors. The role of miR-143 and miR-145 in regulating KRAS expression was already described, however it was never described miR-30c targeting KRAS.

### miR-30c Regulates KRAS Expression

**Figure 4:**
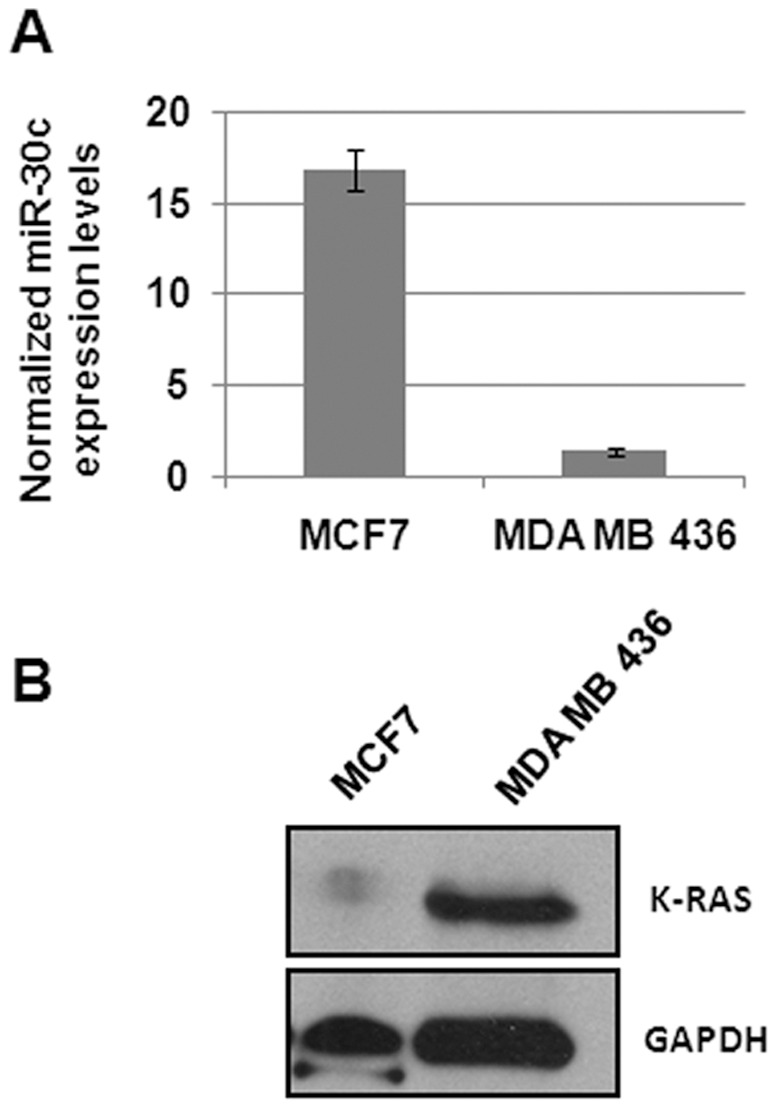
Correlation of expression of miR-30c and KRAS in two breast cancer cell lines. Inverse correlation between the expression level of miR-30 determine by qRT-PCR (A) and detection of KRAS protein in MCF7 and MDA-MB-436 cells (B).

**Figure 5:**
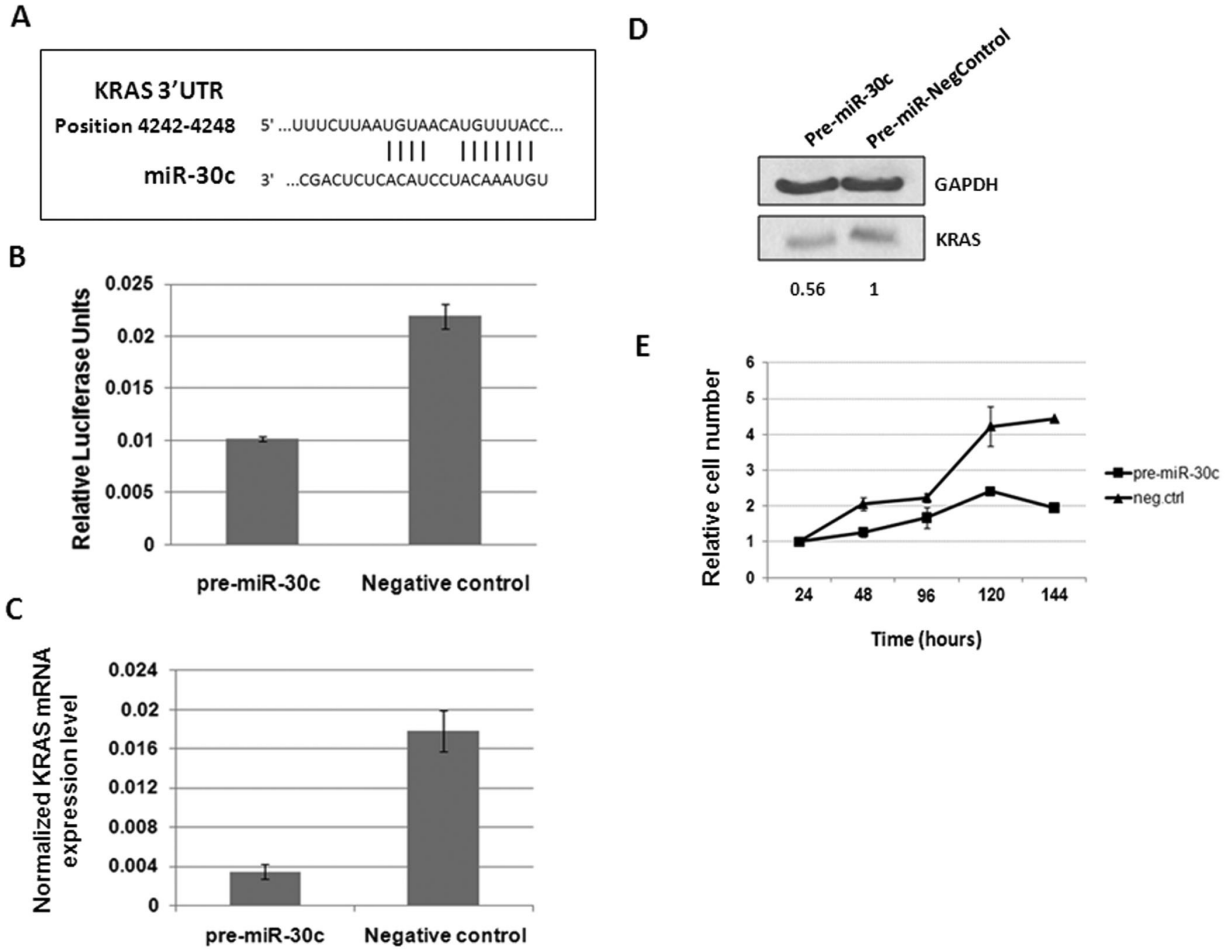
miR-30c effects on KRAS expression and cell proliferation. (A) Schematic representation of miR-30c binding site within the KRAS 3′UTR region. (B) Luciferase activity of a reporter construct carrying the KRAS 3′UTR downstream of the luciferase gene. The construct was co-transfected with pre-miR-30c or scramble control in MDA-MB-436 cells. (C) KRAS expression at transcription level. Significant reduced level of KRAS mRNA expression was detected by qRT-PCR after pre-miR-30c transfection, comparing with scramble control. (D) Regulation of KRAS protein level by miR-30c. MDA-MB-436 cells were transfected with pre-miR-30c or pre-miR-scramble oligonucleotides. After 48 hours KRAS protein was evaluated by western blot. GAPDH was used as loading control. The signal in each line was quantified and the ratio of KRAS to GAPDH was determined. (E) Effect of miR-30c expression on proliferation of MDA-MB-436 cells. MTT cell viability assay was performed at 48, 72, 96, 120 or 144 hours after transfection of MDA-MB-436 cells with pre-miR-30c or pre-miR-scramble oligonucleotides.

We explored the role of miR-30c in regulation of KRAS expression. A negative correlation between miR-30c expression and KRAS protein level was observed in two breast cancer cell lines (Figure 4). In addition, several bioinformatic target prediction algorithms (DIANA microT4.0, TargetScan, PITA, PicTar, miRANDA) indicated existence of a broadly conserved putative binding site for miR-30c in the 3′UTR of the KRAS gene (Figure 5A). To test the hypothesis that KRAS is a bona fide target of miR-30c, we constructed a reporter plasmid harboring 300 pb of the wild-type 3′UTR region of KRAS flanking miR-30c binding site downstream of the luciferase coding region. MDA-MB-436 cells were co-transfected with luciferase reporter and pre-miR-30c or scramble control. As a result, pre-miR-30c transfected cells showed a marked reduction (52%) of luciferase activity compared to scramble control, confirming the interaction between miR-30c and KRAS 3′UTR binding site (Figure 5B). Next, we checked whether miR-30c could affect KRAS mRNA stability by performing qRT-PCR analysis in MDA-MB-436 cells transiently transfected with either pre-miR-30c or scramble control. Indeed, we observed a sharp decrease in KRAS mRNA levels upon transfections with pre-miR-30c (Figure 5C).

In addition, pre-miR-30c or pre-miR- control were transfected into MDA-MB-436 cells, and we confirmed a reduction of about 44% of KRAS protein level in MDA-MB-436 cells over-expressing miR-30c, in comparison to control (Figure 5D).

### miR-30c Affects Proliferation of Breast Cancer Cells

To gain more insight into the biological effects of miR-30c on breast tumorigenesis and given that KRAS plays a role in regulation of cell proliferation, MDA-MB-436 cells, which previously showed elevated levels of KRAS protein, were transfected with pre-miR-30c or scramble and analyzed for cell growth. As shown in Figure 5E, ectopic expression of miR-30c resulted in reduced proliferation in comparison to scramble control transfected cells. Therefore, modulation of KRAS protein level by miR-30c may explain at least in part, why down-regulation of miR-30c can promote proliferation and contribute to tumorigenesis.

## Discussion

miRNA profiling studies in various tumor types have demonstrated a widespread miRNA deregulation, providing for new insights in tumor biology, improved classification and opening new avenues for development of novel targeted therapies in cancer. In this study we performed miRNA expression profiling in familial breast tumors and normal breast tissue in order to uncover deregulated miRNAs. The normal breast tissue series was obtained from healthy individuals, both from BRCA1/2- mutation carriers belonging to high risk families, and from mutation-free individuals undergoing breast reduction surgery. This has enabled us to pinpoint miRNAs important for tumorigenesis, by removing the bias induced through effects of BRCA1/2 mutation on miRNA expression. We have defined down-regulation for 17 miRNAs and up-regulation of miR-21 and miR-300 in familial breast tumors when comparing to normal breast tissue.

A high proportion of deregulated miRNAs in hereditary tumors were commonly found in previous studies with sporadic breast tumors, suggesting that there are miRNAs that likely regulate important oncogenes involved in both familial and sporadic tumors, irrespective of their genetic background. It was expected that comparing tumor samples with normal tissue, which is characterized by quiescent cells, would uncover miRNAs that may be mainly involved in proliferation. Expression analysis of the 19 differentially expressed miRNAs in HMEC cells, which represent normal proliferating cells, have allowed finding some miRNAs overexpressed in normal tissues that were not overexpressed in normal HMEC cells, such as miR-99a, miR-101 or miR-145, which could have a role in the control of proliferation processes in general. Other miRNAs expressed by both HMEC and normal breast and down-regulated in tumors tissue may be involved in other signaling pathways related to tumoral behavior.

Moreover, the pathway enrichment analysis suggested that co-expressed miRNAs seem to collectively target a broad range of signaling pathways related to proliferation and cell migration/motility. Altered cell signaling has long been recognized as a mechanism employed by cells in the development and progression of cancer [18]. Importantly, 20 genes within MAPK signaling pathway were significantly associated with our set of deregulated miRNAs, suggesting that the inhibition of these miRNAs would result in a concomitant activation of MAPK signaling.

In breast cancer, MAPKs play a key role in transducing growth signals from the extracellular environment [19], [20]. The activation of the KRAS/MAPK pathway generates a plethora of responses in breast cancer tumors and cell lines, affecting cell growth, proliferation, differentiation and transformation [21]. In breast cancer development, up-regulation of the KRAS/MAPK signaling can occur through multiple facets, and it has been shown to be increased in many breast cancer samples either by over-expression of growth-factor-receptor tyrosine kinases primarily HER2/ErbB-2, EGFR, and IGFR or by activating mutations [22], [23], [24], [25]. Although KRAS is frequently mutated in human cancers including pancreatic, colorectal and lung cancers, KRAS mutations are extremely rare in breast cancer [26], [27]. However wild-type KRAS is significantly activated in breast cancers that over-express EGFR and ErbB2 [28]. Additionally many investigators have reported over-expression of the KRAS-encoded p21 proteins in breast malignancies in comparison to normal breast tissue [29], [30] although the role of this over-expression in breast carcinogenesis has not been determined.

Interestingly, KRAS was found to be a target of multiple miRNAs found to be down-regulated in breast tumors. The let-7 family of miRNAs has been shown to regulate multiple oncogenes, including KRAS and c-MYC [16], and miR-143/145 are involved in feed-forward mechanism that potentiates Ras signaling through down-regulation of KRAS and Ras-responsive element-binding protein (RREB1), which represses the miR-143/145 promoter [31]. Here we have identified a novel broadly conserved miRNA, miR-30c, as a direct negative regulator of KRAS expression. Interestingly, miR-30 and let-7 were reported to be markedly reduced in breast tumor-initiating cells and contribute to their self-renewal capacity and undifferentiated state, and ectopic expression of these miRNAs in breast tumor-initiating cell xenografts decreases their tumorigenic and metastatic potential [32]. Furthermore, it has been shown recently that higher expression of miR-30c was significantly associated to benefit of tamoxifen treatment and with longer progression-free survival [33]. Altogether, decreased expression of these miRNAs may release the negative regulation of KRAS. Interestingly, our results showed that at least three KRAS regulating miRNAs (miR-30c, miR-143, and miR-145) had significantly reduced co-expression in tumors and then these miRNAs may act together in the regulation of KRAS oncogene.

Signal transduction pathways integrate signals from extracellular stimuli including mitogens, growth factors, hormones and environmental stresses- signals required for tumorigenesis. miRNA deregulation results in the complex modulation of multiple targets belonging to multiple pathways. Commonly deregulated miRNAs in both familial and sporadic breast cancer suggest that commonly altered pathways could be important for tumor progression. Here, we have demonstrated that KRAS inhibition through direct regulation by miR-30c leads to reduced proliferation in breast cancer cells. Similarly, other studies have identified KRAS as a target of several miRNAs down-regulated in tumors (let-7, miR-96 and miR-143), that also have an effect on cancer cell proliferation and tumor invasiveness [15], [16], [17]. Therefore, coordinated down-regulation of miRNAs found in breast tumors would be not only affecting KRAS oncogene expression but also may be targeting other genes of the KRAS/MAPK signaling pathway to cooperatively activate tumorigenic downstream signals.

In general a strong similarity between deregulated miRNAs was found in hereditary and sporadic breast cancer when compared to normal breast tissue. However, in order to get miRNAs associated with BRCA1 and BRCA2 mutated tumors, a higher number of BRCA1/2 mutated tumors would be needed. In this regard, one study found very similar miRNA expression profiles in high grade serous ovarian carcinomas with or without BRCA1/2 mutations [34]. More studies are guaranteed to determine the role of miRNA more related to familial breast tumors and those specifically associated to the BRCA1 and BRCA2 mutated tumors.

In summary, our data defined a deregulated set of miRNAs in hereditary breast tumors, many of them commonly deregulated in sporadic breast cancer. These miRNAs mostly showed significant reduced expression in tumors comparing to normal breast tissue. One of these miRNAs, miR-30c, potentially contributes to breast malignancy formation through release of KRAS suppression suggesting that this miRNA, and likely other miRNAs also targeting KRAS/MAPK signaling, may function as tumor suppressors in breast cancer.

## Materials and Methods

### Ethics Statement

Informed written consent was obtained from all patients involved in this study to perform genetic studies and to use exceeding material for research, and the research project has the approval of the ethics committee of the Spanish National Cancer Research Centre (CNIO), named *Comité de ética de la investigación y de bienestar animal del Instituto de Salud Carlos III.*

### Samples

Tumor tissue samples were obtained from patients undergoing surgery for breast cancer from different Hospitals in Spain. Potential differences regarding ethnicity was not affecting in this case since all tumors were obtained from Spaniards patients. All patients belong to high-risk families with at least three members affected with breast and/or ovarian cancer and at least one of whom was younger than 50 years when diagnosed. For microarray analysis we included whole tissue sections from 22 frozen hereditary breast tumors consisting of 3 BRCA1-mutated, 5 BRCA2 and 14 non-BRCA1/2 (BRCAX) samples, and 14 normal breast tissues including 3 from BRCA1-mutation carriers, 5 from BRCA2-mutation carriers. Histopatological features of the tumors are shown in Table S2, 1 normal breast tissue from contra-lateral breast of patient with BRCAX tumor, and 5 normal breast tissues. Normal breast tissues were obtained after breast reduction surgery from healthy individuals with no family history of breast cancer, and normal breast tissue from BRCA1/2-mutation carriers were obtained after prophylactic surgery. The tissue collection used for validation included 18 paired samples from fresh sporadic breast tumors and their adjacent normal breast tissue counterparts, RNA from 6 additional and 4 normal breast tissue samples was also used for validation. In addition, expression of miR-30c expression was analyzed by qRT-PCR using RNA from FFPE tumor (12 hereditary and 8 sporadic breast tumors) and 9 breast tissue samples.

Normal Human Mammary Epithelial Cells (HMEC) (Clonetics) were used to evaluate miRNA expression comparing with normal breast tissue. HMEC cells were grown in MEGM, Mammary Epithelial Growth Medium (Clonetics) supplemented with growth factors SingleQuots (Clonetics) in absence of FBS.

### miRNA Microarray

Total RNA was extracted from primary tumors using Trizol (Invitrogen). RNA quantity and quality were assessed by NanoDrop Spectrophotometer (Nanodrop Technologies, Wilmington, DE, USA) and Agilent 2100 Bioanalyzer (Agilent Technologies, Santa Clara, CA, USA), respectively. Microarray expression profiling was performed using miRCURY LNA™ microRNA Array kit (Exiqon A/S, Denmark) according to manufacturer’s instructions, in a one-color, pair-wise comparison experimental design. Briefly, 300 ng of total RNA was labeled with Hy3 fluorescent dye and subsequently hybridized over 16 h at 56°C onto a miRNA microarray chip (v.11.0– hsa, mmu & rno) containing 1940 capture probes, in 4 replicates, representing 831 human miRNAs annotated in miRBasev.11 database. A set of 10 synthetic Spike-in RNAs was added to total RNA sample prior to labeling and later used for quality control. Processed slides were scanned with Agilent Array scanner (Agilent Technologies), with the laser set to 635 nm, at Power 80 and PMT 70 setting, and a scan resolution of 10 µm. Fluorescence intensities on scanned images were quantified using Feature Extraction software (Agilent Technologies) using the modified Exiqon protocol. Average values of the replicate spots were background subtracted and log transformed and subjected to further analysis. Microarray dataset is publicly available at GEO database http://www.ncbi.nlm.nih.gov/geo/info/linking.html under GEO accession number GSE32922.

### Array Data Processing and Statistical Analysis

Raw data were quantile normalized for inter-array variability. Data was preprocessed to eliminate miRNAs with uniformly low expression or with low expression variation (SD<0.3) across the experiments, retaining 466 miRNA genes (306 hsa-miR +160 hsa-miRPlus). Average linkage hierarchical clustering (Pearson correlation, uncentered metrics) from Gene Cluster and Treeview (http://rana.stanford.edu/software) algorithms were used to obtain clustering of the data sets. To determine if there were genes differentially expressed between tumor and normal breast tissue, differential expression analysis was performed using t-test implemented in the POMELO II tool, available in Asterias web server (http://asterias.bioinfo.cnio.es) [35]. The estimated significance level were obtained by permutation testing and p-values were corrected for multiple hypotheses testing using Benjamini & Hochberg False Discovery Rate (FDR) adjustment [36]. Those miRNAs with FDR <0.05 were selected as significantly differentially expressed.

### Quantitative RT-PCR Analysis

Quantitative RT-PCR analysis of miR-30c, miR-100, miR-125b and miR-320a was performed on independent series of familial tumors as well as sporadic breast tumor samples and compared to normal breast tissue samples using miRCURY LNA™ microRNA PCR System (Exiqon). Briefly, 10 ng of total RNA was reverse-transcribed with miRNA specific primers and Transcriptor Reverse Transcriptase, and the cDNA was used as a template for the qPCR reaction using miRNA specific LNA™ PCR primer and Universal PCR primer. Gene expression levels were quantified using the ABI Prism Sequence Detection System 7900HT (Applied Biosystems). All experiments were performed in triplicate and the mean of triplicates was used. Normalization was done with SNORA66 RNA and 5S rRNA. Relative expression was calculated using the comparative Ct method.

### Pathway Enrichment Analysis

DIANA miRPath pathway enrichment analysis (http://diana.cslab.ece.ntua.gr/) was used to gain insight into global molecular networks and canonical pathways related to differentially expressed miRNAs between normal and tumor samples. DIANA miRPath is a web-based computational tool developed to identify molecular pathways potentially altered by the expression of single or multiple microRNAs. The software performs an enrichment analysis of multiple microRNA target genes comparing each set of microRNA targets to all known KEGG pathways. Those pathways showing p-value <0.05, were considered significantly enriched between classes under comparison.

### Cell Culture, Constructs and Transfections

The MDA-MB-436 was kindly provided by Dr. K.S Massey-Brown from Department of Pharmacology and Toxicology, University of Arizona, Tucson, USA, and was characterized in previous studies by our group [37]. The MDA-MB-436 cell line was cultured in RPMI 1640 medium supplemented with 10% FBS and 100 units/ml of Penicillin G and streptomycin. Cells were split 24 h prior to all transfection assays at a confluence 40–70%. Pre-miRNA oligonucleotides (pre-miR-30c and scramble control) were purchased from Ambion (Austin, Texas, USA). For luciferase reporter assay transfection of oligonucleotides and plasmids was performed using Lipofectamine 2000 according to the protocols provided by the manufacturer (Invitrogen, Calsbad, CA). For proliferation, and KRAS western blot analysis, pre-miRNA oligonucleotides were transfected to a 50 nM final concentration using Oligofectamine (Invitrogen).

### KRAS-3′UTR Luciferase Reporter Assay

3′UTR sequence of the KRAS was retrieved through NCBI nucleotide database. A 300 bp fragment of the 3′UTR region of KRAS gene containing miR-30c binding site, was amplified by PCR from human genomic DNA, and cloned into a modified pGL3-Control vector (Promega) at the SacII and EcoRI site, immediately downstream of the luciferase stop codon. Primer sequences used to amplify this region were RAS3UTR-F: 5′CAC*GAATTC* CACACCCCCACAGAGCTAAC3’ and RAS3UTR-R: 5′TTCCCGCGGTGTTTGATATGACCAACATTCCT 3′. Correct vector construction was verified by direct sequencing. Dual luciferase assay was carried out by co-transfecting MDA-MB-436 cells with 25 pmol of pre-miR-30c or scramble control, along with 500 ng of KRAS 3′UTR- firefly luciferase construct and 7.5 ng of *Renilla* luciferase vector, using Lipofectamine 2000 (Invitrogen) per well, according to the manufacturer’s protocol in a 24-well plate format. Cells were grown for 48 h, after which luciferase activity was assayed with Dual-Luciferase Assay System (Promega). Experiments were performed in triplicate and *Renilla* luciferase activity was used for transfection variation normalization.

### Detection of KRAS mRNA and Protein Levels

To detect gene expression levels of KRAS, one µg of total RNA was reverse transcribed using MMLV Reverse Transcriptase (Invitrogen) and random primers. The cDNAs were subjected to quantitative RT-PCR assay with the use of labeled probes (Roche Universal Probe library) and the TaqMan Universal PCR Mix in an ABI Prism Sequence Detection System 7900HT (Applied Biosystems) under manufacturer’s recommendations. The PCR amplification was carried out with 10 min at 95°C, followed by 50 cycles of 15 s at 95°C and 1 min at 60°C, using the oligonucleotides shown in Table S3. ß-actin was used as internal control and allowed normalization of the samples. All experiments were analyzed in triplicate.

Western blot analysis was performed using standard procedures for whole-cell extracts from cell lines. Lysates were prepared using RIPA buffer (Sigma-Aldrich). Equal amounts of protein lysates (50–100 µg) were separated by SDS-PAGE on 4–12% pre-casted gels (Invitrogen), and electrotransferred to nitrocelulose membranes (Wathman) and probed with primary antibody. Antibodies used were KRAS (F234, Santa Cruz) and GAPDH (CNIO, Monoclonal Antibodies Unit). Secondary antibody staining was carried out with anti-mouse HRP IgG (Dakko) and HPR activity was detected with ECL Detection System (GA Healthcare).

### Cell Proliferation Assay

Cell viability was assessed by using 3-(4,5-dimethylthiazol-2-yl)-2,5-diphenyl tetrazolium bromide (MTT) assay. MDA-MB-436 cells were transfected in independent experiments with pre-miR-30c/pre-miR-Scramble oligonucleotides. Cells were incubated in 1 µg/µl MTT Formazan (Sigma-Aldrich) diluted in normal culture medium at 37°C for 5 h. Cell viability was determined at 24, 48, 72, 96, 120 or 144 hours after transfection.

## Supporting Information

Figure S1 **Validation of miRNA expression by qRT-PCR in hereditary and sporadic tumors.** Expression levels of miR-125b, miR-100, miR-320a in normal breast tissue comparing with familial tumor samples (FamBC) and sporadic breast cancer (SpoBC). Differences were estimated by t-test and *p* values are shown for each case.
(DOC)

Table S1 **Predicted genes within MAPK pathway targeted by deregulated miRNAs in hereditary breast cancer tumors.**
(DOC)

Table S2 **Histopathological data from hereditary breast tumors.**
(DOC)

Table S3 **Primers used for measuring KRAS expression by quantitative RT-PCR (used with Universal ProbeLibrary probe#62, Roche).**
(DOC)
