# Supplementary material for: Deregulated miRNAs in Hereditary Breast Cancer Revealed a Role for miR-30c in Regulating KRAS Oncogene
Source: PLoS One. 2012 Jun 11;7(6):e38847. doi: 10.1371/journal.pone.0038847 (PMC3372467; doi:10.1371/journal.pone.0038847)
Supplement: Figure S1 — Validation of miRNA expression by qRT-PCR in hereditary and sporadic tumors. Expression levels of miR-125b, miR-100, miR-320a in normal breast tissue comparing with familial tumor samples (FamBC) and sporadic breast cancer (SpoBC). Differences were estimated by t-test and p values are shown for each case. (DOC) [file pone.0038847.s001.doc]

**Figure S1. Validation of miRNA expression by qRT-PCR in hereditary and sporadic tumors.** Expression levels of miR-125b, miR-100, miR-320a in normal breast tissue comparing with familial tumor samples (FamBC) and sporadic breast cancer (SpoBC). Differences were estimated by t-test and *p* values are shown for each case.
