# Supplementary material for: Deregulated miRNAs in Hereditary Breast Cancer Revealed a Role for miR-30c in Regulating KRAS Oncogene
Source: PLoS One. 2012 Jun 11;7(6):e38847. doi: 10.1371/journal.pone.0038847 (PMC3372467; doi:10.1371/journal.pone.0038847)
Supplement: Table S1 — Predicted genes within MAPK pathway targeted by deregulated miRNAs in hereditary breast cancer tumors. (DOC) [file pone.0038847.s002.doc]

**Table S1. Predicted genes within MAPK pathway targeted by deregulated miRNAs in hereditary breast cancer tumors.**

| **miRNA Name** | **Gene** | **Gene Definition** |
| --- | --- | --- |
| hsa-miR-205 | RPS6KA3 | ribosomal protein S6 kinase, 90kDa, polypeptide 6 |
| hsa-miR-99a | PPP3CA | calcium binding protein P22 |
|  | FGFR1 | fibroblast growth factor receptor 1 |
| hsa-miR-100 | PPP3CA | calcium binding protein P22 |
|  | FGFR1 | fibroblast growth factor receptor 1 |
| hsa-miR-195 | FGF2 | fibroblast growth factor 2 |
|  | MAPK8 | mitogen-activated protein kinase 8 |
|  | AKT3 | v-akt murine thymoma viral oncogene homolog 3 (protein kinase B, gamma) |
| hsa-miR-101 | RAP1B | RAP1A, member of RAS oncogene family |
|  | STK4 | serine/threonine kinase 3 |
| hsa-miR-130a | RPS6KA5 | ribosomal protein S6 kinase, 90kDa, polypeptide 6 |
| hsa-miR-300 | PPP3CA | calcium binding protein P22 |
|  | MEF2C | myocyte enhancer factor 2C |
| hsa-miR-30c | SOS1 | son of sevenless homolog 1 (Drosophila) |
|  | KRAS | v-Ki-ras2 Kirsten rat sarcoma viral oncogene homolog |
|  | NF1 | neurofibromin 1 |
|  | RASA1 | RAS p21 protein activator (GTPase activating protein) 1 |
|  | RAP1B | RAP1A, member of RAS oncogene family |
|  | PPP3CA | calcium binding protein P22 |
|  | MAPK8 | mitogen-activated protein kinase 8 |
|  | MAP4K4 | mitogen-activated protein kinase kinase kinase kinase 4 |
|  | MAP3K1 | mitogen-activated protein kinase kinase kinase 1 |
|  | TAOK1 | TAO kinase 3 |
|  | BDNF | brain-derived neurotrophic factor |
|  | CRKL | v-crk sarcoma virus CT10 oncogene homolog (avian) |
