# Supplementary material for: Deregulated miRNAs in Hereditary Breast Cancer Revealed a Role for miR-30c in Regulating KRAS Oncogene
Source: PLoS One. 2012 Jun 11;7(6):e38847. doi: 10.1371/journal.pone.0038847 (PMC3372467; doi:10.1371/journal.pone.0038847)
Supplement: Table S2 — Histopathological data from hereditary breast tumors. (DOC) [file pone.0038847.s003.doc]

**Table S2. Histopathological data from hereditary breast tumors.**

|  |  |  |  |  |  |  |  |  |
| --- | --- | --- | --- | --- | --- | --- | --- | --- |
| Number | ID | Germline mutation | Lymph node status | Grade | ER | PR | HER2 | Subtype |
| 1 | 01T163 | BRCA1 | Positive | 3 | - | - | - | Basal |
| 2 | 01T167 | BRCA1 | Negative | 3 | - | - | - | Basal |
| 3 | 01T185 | BRCA1 | Positive | 3 | - | - | - | Basal |
| 4 | 02T124 | BRCA1 | Negative | 3 | - | - | - | Basal |
| 5 | 02T144 | BRCA1 | NA | NA | NA | NA | NA | NA |
| 6 | 05T126 | BRCA1 | NA | 3 | - | - | - | Basal |
| 7 | 01T265 | BRCA2 | NA | NA | NA | NA | NA | NA |
| 8 | 05T312 | BRCA2 | Positive | 2 | + | - | - | Luminal A |
| 9 | 09T155 | BRCA2 | NA | NA | NA | NA | NA | NA |
| 10 | 01T248 | BRCAX | NA | NA | + | + | + | Luminal B |
| 11 | 01T306 | BRCAX | Negative | 1 | + | + | - | Luminal A |
| 12 | 02T328 | BRCAX | Negative | 3 | - | - | + | Her2+ |
| 13 | 04T59 | BRCAX | NA | NA | NA | NA | NA | NA |
| 14 | 05T129 | BRCAX | Negative | 2 | + | - | + | Luminal B |
| 15 | 05T132 | BRCAX | NA | NA | NA | NA | NA | NA |
| 16 | 05T134 | BRCAX | NA | 3 | + | - | - | Luminal A |
| 17 | 05T136 | BRCAX | Negative | 1 | + | + | - | Luminal A |
| 18 | 09T154 | BRCAX | NA | NA | NA | NA | NA | NA |
| 19 | 09T79 | BRCAX | Negative | 2 | - | - | + | Her2+ |

NA: Not available data.
