# Supplementary material for: Deregulated miRNAs in Hereditary Breast Cancer Revealed a Role for miR-30c in Regulating KRAS Oncogene
Source: PLoS One. 2012 Jun 11;7(6):e38847. doi: 10.1371/journal.pone.0038847 (PMC3372467; doi:10.1371/journal.pone.0038847)
Supplement: Table S3 — Primers used for measuring KRAS expression by quantitative RT-PCR (used with Universal ProbeLibrary probe#62, Roche). (DOC) [file pone.0038847.s004.doc]

**Table S3. Primers used for measuring KRAS expression by quantitative RT-PCR (used with Universal ProbeLibrary probe#62, Roche).**

| **Primer** | **Sequence (5’-3’)** |
| --- | --- |
| KRAS_F | tggacgaatatgatccaacaat |
| KRAS_R | tccctcattgcactgtactcc |
